# Early Discovery of Children With Lysosomal Acid Lipase Deficiency With the Universal Familial Hypercholesterolemia Screening Program

**DOI:** 10.3389/fgene.2022.936121

**Published:** 2022-07-12

**Authors:** Ursa Sustar, Urh Groselj, Katarina Trebusak Podkrajsek, Matej Mlinaric, Jernej Kovac, Martin Thaler, Ana Drole Torkar, Ajda Skarlovnik, Tadej Battelino, Tinka Hovnik

**Affiliations:** ^1^ Department of Endocrinology, Diabetes and Metabolic Diseases, University Children’s Hospital, University Medical Centre Ljubljana, Ljubljana, Slovenia; ^2^ Faculty of Medicine, University of Ljubljana, Ljubljana, Slovenia; ^3^ Department of Medicine, Division of Cardiovascular Medicine, Stanford University, Stanford, CA, United States; ^4^ Clinical Institute of Special Laboratory Diagnostics, University Children’s Hospital, University Medical Centre Ljubljana, Ljubljana, Slovenia; ^5^ Faculty of Medicine, Institute of Biochemistry and Molecular Genetics, University of Ljubljana, Ljubljana, Slovenia; ^6^ Department of Radiology, University Children’s Hospital Ljubljana, Ljubljana, Slovenia; ^7^ Department of Vascular Diseases, University Medical Centre Ljubljana, Ljubljana, Slovenia

**Keywords:** cholesterol ester storage disease, CESD, lysosomal acid lipase deficiency, LAL-D, *LIPA* gene, hypercholesterolemia, universal screening, pediatric population

## Abstract

Lysosomal acid lipase deficiency (LAL-D) is an autosomal recessive lysosomal storage disorder, caused by homozygous or compound heterozygous pathogenic variants in the *LIPA* gene. Clinically, LAL-D is under- and misdiagnosed, due to similar clinical and laboratory findings with other cholesterol or liver misfunctions. As a part of the Slovenian universal familial hypercholesterolemia (FH) screening, LAL-D is screened as a secondary condition among other rare dyslipidemias manifesting with hypercholesterolemia. Out of 669 children included, three were positive for a homozygous disease-causing splicing variant NM_000235.4: c.894G > A (NP_000226.2:p. Gln298Gln) in the *LIPA* gene (NG_008194.1). The mean age by the diagnosis of LAL-D was 9.8 ± 0.9 years. Moreover, all three LAL-D-positive children had an important elevation of transaminases and decreased activity of the lysosomal acid lipase enzyme. Abdominal MRI in all children detected an enlarged liver but a normal-sized spleen. In conclusion, universal FH screening algorithms with the confirmatory genetic analysis in the pediatric population enable also rare dyslipidemia detection at an early age. An important clinical criterion for differentiation between FH and the LAL-D-positive children has elevated transaminase levels (AST and ALT). In all three LAL-D positive children, an improvement in cholesterol and transaminase levels and steatosis of the liver has been seen after early treatment initiation.

## Introduction

Lysosomal acid lipase deficiency (LAL-D) is an autosomal recessive lysosomal storage disorder, caused by homozygous or compound heterozygous disease-causing variants in the *LIPA* gene located on chromosome 10q23.2 (Pisciotta et al., 2017; Wilson and Patni, 2020). Depending on the genetic variant in the *LIPA* gene and consequently residual lysosomal acid lipase (LAL) activity, LAL-D can result in the very severe, infantile-onset, and lethal form known as Wolman disease, or in the milder, late-onset phenotype, known also as cholesteryl ester storage disease (CESD) (Reiner et al., 2014; Pullinger et al., 2015; Sjouke et al., 2016; Maciejko, 2017).

In CESD, various symptoms are present and usually begin in midchildhood with an average age of 5 (Maciejko, 2017), although they can develop later in adulthood. Most of the affected children present symptoms, such as elevated low-density lipoprotein cholesterol (LDL-C) levels, low high-density lipoprotein cholesterol (HDL-C) levels, and accelerated atherosclerotic cardiovascular disease (CVD) hepatomegaly, and splenomegaly, gradually leading to liver fibrosis and cirrhosis. Moreover, some children suffer from malabsorption, vomiting, diarrhea, steatorrhea, and growth failure (Reiner et al., 2014; Pullinger et al., 2015; Sjouke et al., 2016; Maciejko, 2017).

Clinically, LAL-D is under- and misdiagnosed, due to similar clinical and laboratory findings with familial hypercholesterolemia (FH) or nonalcoholic fatty liver disease (NAFLD), leading to a delay in treatment or mistreatment. A correct diagnosis of LAL-D is critical for the appropriate clinical management of children (Pullinger et al., 2015). The standard way of the LAL-D diagnosis is a demonstration of decreased LAL enzyme activity in DBS, serum, or skin fibroblasts. Elevated liver transaminases and elevated serum TC or LDL-C could raise the suspicion of the LAL-D diagnosis (Pullinger et al., 2015; Maciejko, 2017). Most cases are diagnosed in the first two decades of life (Muntoni et al., 2007; Bernstein et al., 2013; Burton et al., 2015). Usage of a human recombinant LAL (sebelipase alpha, Kanuma^®^) for the LAL-D treatment was approved in 2015 resulting in significant improvements in the hepatic and lipid profiles of children with LAL-D (Sjouke et al., 2016; Camarena et al., 2017).

The prevalence of the Wolman disease is estimated at approximately 1/350,000, whereas the prevalence of the CESD is estimated between 1/40,000 and 1/300,000, depending on ethnicity and geographic location. However, due to milder phenotypes of the CESD and overlap with other more frequent pathologies, the prevalence might be underestimated (Pullinger et al., 2015; Camarena et al., 2017).

In the study, we assessed the adoption of universal FH screening for early LAL-D detection in preschool children. Furthermore, we evaluated clinical criteria for differentiation between FH and the LAL-D-positive children. Moreover, we presented clinical characteristics of the LAL-D-positive children.

## Methods

### Cohort Description

Slovenia has been implementing universal FH screening in children since 1995 as a routinely part of the blood checkup at the programmed visit of all 5-year-old children to the primary care pediatrician (Sedej et al., 2014; Klancar et al., 2015; Groselj et al., 2018, 2022), lately reaching more than 90% of 5-year-old children (of approximately 20,000) each year (Supplementary Figure S1). As a part of the universal FH screening program total cholesterol (TC) was measured. In 2011, routine genetic diagnostic for the FH-related genes was introduced at the University Children’s Hospital Ljubljana, Clinical Institute of Special Laboratory Diagnostics (Klancar et al., 2015; Groselj et al., 2018; Marusic et al., 2020). According to the clinical guidelines, additional cascade screening of family members and further clinical care are performed as required and financed by the Slovenia’s national health insurance system.

As the patients with LAL-D also have elevated TC levels, they are detected by our universal FH screening and referred to the Universal Children’s Hospital Ljubljana. Since 2018, in our expended NGS (next-generation sequencing) panel, other genes also associated with dyslipidemia are included. The *LIPA* gene is also one of those included on the NGS panel. Until recently, 669 children had the *LIPA* gene sequenced. For the children referred as a part of the FH screening before applying the expended NGS panel (prior 2018), who were negative for the FH-related genes and had elevated aspartate aminotransferase – AST (>0.58 μkat/L) – and/or alanine aminotransferase – ALT levels (>0.74 μkat/L), Sanger sequencing for the *LIPA* gene was additionally performed. After 2018, all FH screening positive children were with the FH-related genes simultaneously also tested for the pathogenic variants in the *LIPA* gene. A flowchart of the included children is presented in Supplementary Figure S2.

The principles of the Declaration of Helsinki were followed, and the Slovenian National Medical Ethics Committee approved the study (#25/12/10, #63/07/13, 0120-14/2017/5, 0120-273/2019/9 and 0120-273/2019/19). Written informed consent was obtained from all parents or legal guardians.

### Genetic Analyses

Genomic DNA was isolated from the whole blood samples of 669 children using the FlexiGene isolation kit (Qiagen, Germany). Two different sequencing methods for *LIPA* gene sequencing were used over time: targeted Sanger sequencing (*n* = 28) and xGen^®^ Lockdown^®^ NGS Probes (IDT, United States) (*n* = 641). Targeted Sanger sequencing was applied for FH-negative children with elevated aspartate aminotransferase – AST (>0.58 μkat/L) – and/or alanine aminotransferase – ALT levels (>0.74 μkat/L), and for the cascade LAL-D screening of the positive siblings. With the usage of the NGS probes, the *LIPA* gene was included in our expanded dyslipidemia gene panel. Samples were sequenced on the MiSeq sequencer with MiSeq Reagent Kit (Illumina, United States) following the manufacturer’s instructions including recommendations for quality control parameters. All variants identified with NGS sequencing were reconfirmed by targeted Sanger DNA sequencing. The pathogenicity of the identified variants was assessed using Human Gene Mutation Database Professional and ClinVar (Landrum et al., 2018) databases. For variants of unknown clinical significance *in silico* prediction tools (CADD, SIFT, MutationTaster, PolyPhen2) were utilized (Ng and Henikoff, 2003; Schwarz et al., 2010; Adzhubei et al., 2013; Rentzsch et al., 2019). Variants were classified according to the American College for Medical Genetics and Genomics (ACMG) classification criteria (Richards et al., 2015).

### Laboratory Findings, Medical Imaging and Pathology Evaluation

Lipid profiles [TC, LDL-C, HDL-C, and triglycerides (TG)] for all the included children were routinely measured with an automated analyzer Advia 1800 (Siemens Healthcare, Erlangen, Germany), using the direct enzymatic colorimetric method. Additionally, transaminase [AST, ALT, and amma-glutamyl transferase (GGT)] levels were measured. South Glasgow Hospital performed acid lipase enzyme assays for the LAL-D-positive children with a pathogenic variant in the *LIPA* gene. Liver MRI elastography and liver ultrasound were performed in the LAL-D-positive children. In two patients, liver biopsy was also performed before considering treatment with sebelipase alpha (Kanuma^®^).

### Arterial Stiffness and Endothelial Function Evaluation

The ultrasound examination was performed using the Aloka 5500 SSD Pro-sound ultrasound machine. The UST-5539 7.5 linear probe was used for vascular examination, and the auto-IMT modality of ultrasound machine software was used. The maximum cIMT value, expressed in millimeters, was used for analysis and determined as the mean of all six measurements performed bilaterally. Using a high-definition echo-tracking system (Aloka implemented E-tracking software) the pressure–diameter curve of the artery was derived and from the time delay between the two adjacent distension waveforms, one-point local pulse wave velocity and beta stiffness was calculated.

The endothelial function measurement was performed on the peripheral arterial tone using the EndoPAT 2000 device (Itamar Medical Ltd., Caesarea, Israel); the reactive hyperemia index (RHI) was calculated using the manufacturer’s algorithm based on the ratios between pulse wave amplitudes during the reactive hyperemia and baseline phases.

### Prevalence Estimation of Heterozygous *LIPA* Variants in the General Population

The prevalence of LAL-D in the Slovenian population was determined based on the cohort who did not express FH phenotype (*n* = 1,915). The number of heterozygous carriers of the pathogenic alleles in the *LIPA* gene was determined, and the prevalence of homozygous LAL-D in our population was then calculated with the Hardy–Weinberg equation.

## Results

### LAL-D-Positive Children Diagnosis

To date, 669 children (demographical data of the cohort is presented in Table 1) were included in the *LIPA* gene analysis. 664 of them were referred to our clinic because of elevated cholesterol levels found at the universal FH screening in children program in Slovenia; four children were referred as symptomatic for LAL-D and one as a part of a sibling-cascade LAL-D screening. Due to the FH screening program, the median age at the first appointment at our institution was 6.3 (IQR: 5.8–7.2) years.

**TABLE 1:** Demographical data of the children included in the study.

		M (N = 269)	F (N = 398)	Total (N = 667)
At first examination at our clinic				
Age		6.4 (5.8, 7.4)	6.3 (5.8, 7.1)	6.3 (5.8, 7.2)
Weight	kg	24.4 (21.3, 29.6)	22.8 (20.1, 27.5)	23.4 (20.5, 28.4)
Height	cm	122.0 (117.0, 130.3)	120.5 (115.5, 127.0)	121.2 (116.0, 128.5)
LDL-C	mmol/L	3.4 (2.9, 3.9)	3.5 (3.1, 4.1)	3.5 (3.0, 4.1)
	mg/dL	131.5 (112.1, 150.8)	135.3 (119.9, 158.5)	135.3 (116.0, 158.5)
TC	mmol/L	5.5 (5.0, 5.9)	5.6 (5.1, 6.1)	5.5 (5.0, 6.0)
	mg/dL	212.7 (193.4, 228.2)	216.6 (197.2, 235.9)	212.7 (193.4, 232.0)
HDL-C	mmol/L	1.5 (1.3, 1.7)	1.5 (1.3, 1.8)	1.5 (1.3, 1.8)
	mg/dL	58.0 (50.3, 65.7)	58.0 (50.3, 69.6)	58.0 (50.3, 69.6)
TG	mmol/L	0.9 (0.7, 1.4)	0.9 (0.7, 1.3)	0.9 (0.7, 1.3)
	mg/dL	79.7 (62.0, 124.0)	79.7 (62.0, 115.1)	79.7 (62.0, 115.1)
AST	µkat/L	0.5 (0.4, 0.6)	0.5 (0.4, 0.6)	0.5 (0.4, 0.6)
ALT	µkat/L	0.3 (0.3, 0.4)	0.3 (0.3, 0.4)	0.3 (0.3, 0.4)

Age, weight, height, and lipid profile of the cohort were included in the LIPA sequencing, at the first examination at our clinic. Data are presented as median (IQR). LDL-C, low-density lipoprotein cholesterol; TC, total cholesterol; HDL-C, high-density lipoprotein cholesterol; TG, triglycerides; AST, aspartate transaminase; ALT, alanine transaminase.

Of all the 669 children analyzed for the *LIPA* and FH-related genes (*APOB*, *LDLR*, and *PCSK9*), 189 were classified as positive or with a variant of uncertain significance (VUS) for the FH-related genes. From the remaining group of 480 children, three were classified as LAL-D-positive. All of them were homozygous for NM_000235.4:c.894G > A variant in the *LIPA* gene (NG_008194.1) resulting in NP_000226.2:p. Gln298Gln (rs116928232) synonymous protein variant affecting the splice site at the end of exon 8. According to the ACMG criteria, the variant was classified as pathogenic (PP5, PP3, PP1, and PM2) (Richards et al., 2015). Two children were detected directly based on the universal FH screening program and one based on the cascade LAL-D screening of siblings. None of the children who were referred as symptomatic for LAL-D (with elevated aminotransferase levels) was positive for a pathogenic variant in the *LIPA* gene. The mean age of the diagnosis was 9.8 ± 0.9 years. Demographical and clinical characteristics of the LAL-D-positive children are presented in Table 2. There was no evidence of consanguineous marriage in the family.

**TABLE 2:** Demographical and clinical characteristics of the LAL-D positive patients.

	Patient 1	Patient 2	Patient 3
At diagnosis			
Age (years)	10.3	8.7	10.3
Screening type	Cascade	Universal	Universal
At first examination at our clinic			
Age (years)	5.8	7.0	5.8
Weight [kg and (percentile)]	19.3 (39.4)	19.2 (10.3)	17.4 (9.5)
Height [cm and (percentile)]	109.2 (15.2)	114.1 (8.3)	108.5 (9.7)
BMI [kg/m^2^ and (percentile) ]	16.2 (67.2)	14.8 (27.9)	14.8 (27.5)
LDL-C (mmol/L)	3.6	4.5	5.3
LDL-C (mg/dL)	139.2	174.0	204.9
TC (mmol/L)	5.3	6.4	7.1
TC (mg/dL)	204.9	247.5	274.6
HDL-C (mmol/L)	1.2	1.5	1.3
HDL-C (mg/dL)	46.4	58.0	50.3
TG (mmol/L)	1.2	0.9	1
TG (mg/dL)	106.3	79.7	88.6
AST (µkat/L)	1.8	1.3	1.4
ALT (µkat/L)	2.3	1.6	1.4

Age at diagnosis, screening type, and lipid profile is represented for the LAL-D-positive patients. BMI, body mass index; LDL-C, low-density lipoprotein cholesterol; TC, total cholesterol; HDL-C, high-density lipoprotein cholesterol; TG, triglycerides; AST, aspartate transaminase; ALT, alanine transaminase.

In all three LAL-D positive children, the enzyme activity of LAL performed from a sample of dried blood spot showed a decreased activity of under 0.02 nmol/punch/hour (reference range: 0.37–2.30 nmol/punch/hour). In patient 3, a liver biopsy was performed before the genetic result was available and showed small droplet steatosis in hepatocytes and rarely captured macrophages. A mildly multiplied and condensed connective tissue in the portal field, in the sinusoids and segmentally in the bile ducts and between hepatocytes, was present. No cholesterol needles were found. Also, a liver biopsy was performed in patient 1 before consideration for the Kanuma treatment, but a milder phenotype was present, and patient 1 was not eligible for the treatment.

### Clinical Follow-Up of the LAL-D-Positive Children

**FIGURE 1:**
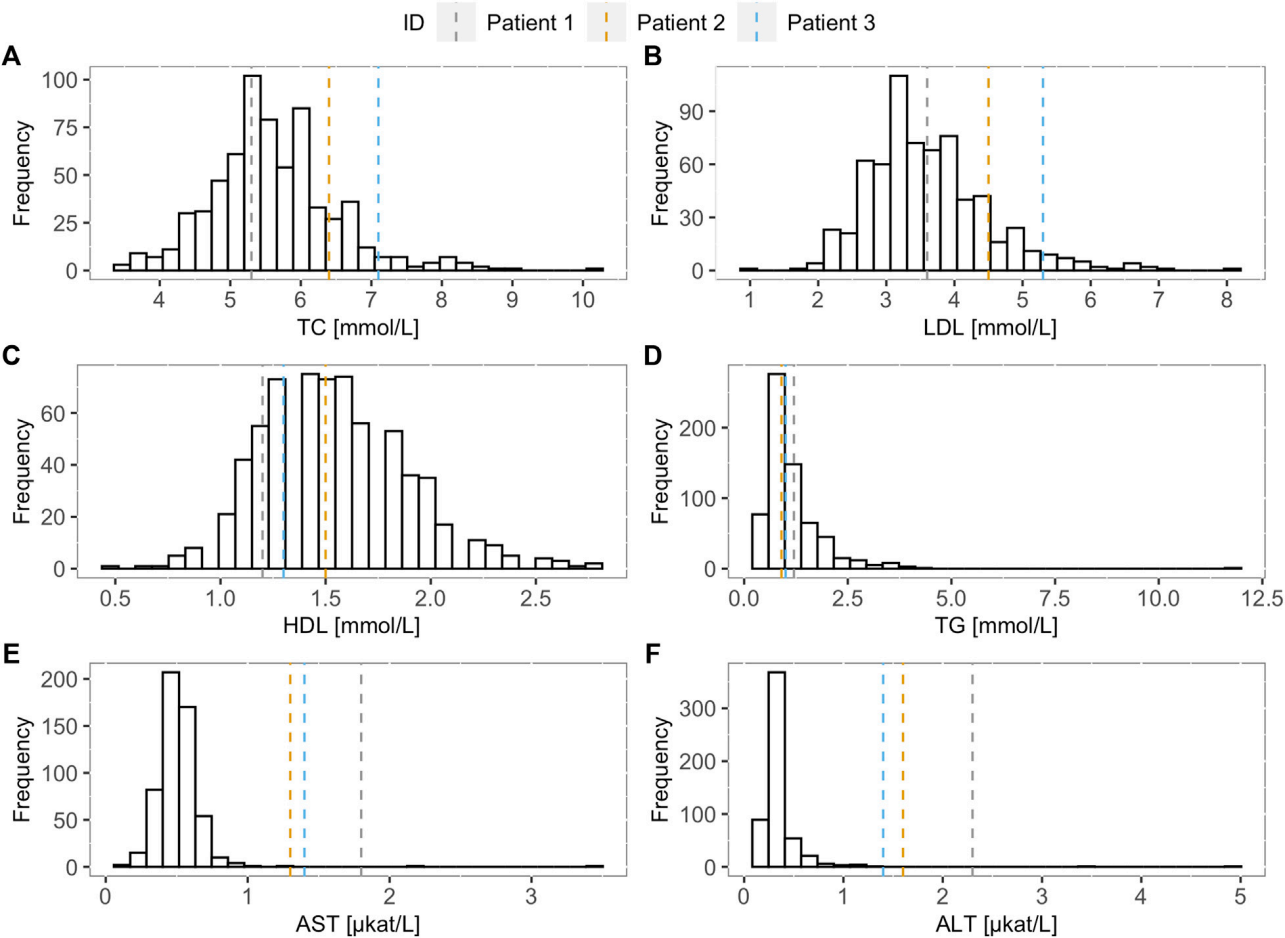
Lipoprotein and transaminase levels of the LAL-D positive children compared to the LAL-D negative cohort. Distribution of **(A)** total cholesterol, **(B)** low-density lipoprotein cholesterol, **(C)** high-density lipoprotein cholesterol, **(D)** triglycerides, **(E)** aspartate transaminase, and **(F)** alanine transaminase for LAL-D negative population. The vertical dotted lines show the values of each parameter for the LAL-D-positive patients. LDL-C: low-density lipoprotein cholesterol; TC: total cholesterol; HDL-C: high-density lipoprotein cholesterol; TG: triglycerides; AST: aspartate transaminase; ALT: alanine transaminase.

When comparing TC, LDL-C, HDL-C, TG, AST, and ALT levels of the FH screening positive children and the LAL-D-positive children, increase in TC and LDL-C levels can be observed in LAL-D-positive children versus the median of the FH screening positive children group (Figure 1) at their first visit at our clinic. Moreover, all three LAL-D-positive children had an important elevation of transaminases.

**FIGURE 2:**
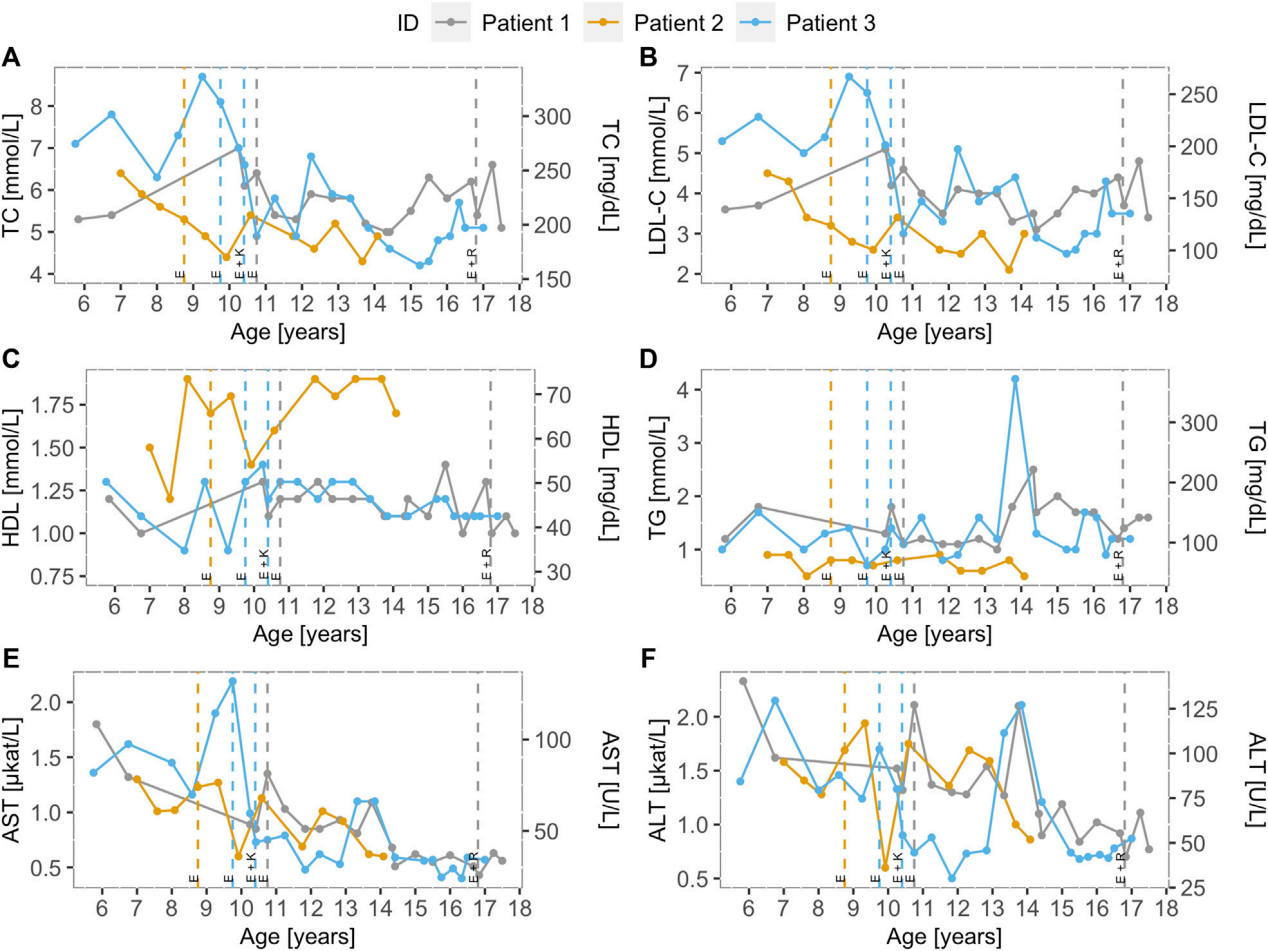
Lipoprotein and transaminase levels of the LAL-D positive children over time. Distribution of **(A)** total cholesterol, **(B)** low-density lipoprotein cholesterol, **(C)** high-density lipoprotein cholesterol, **(D)** triglycerides, **(E)** aspartate transaminase, and **(F)** alanine transaminase for LAL-D-positive population over time. Dotted vertical lines represent the beginning of the treatment and treatment modification for each patient. All three patients were treated with ezetimibe (10 mg). Furthermore, patient 3 was treated with a combination of ezetimibe (10 mg) and Kanuma® and patient 1 was treated with a combination of ezetimibe (10 mg) and rosuvastatin (10 mg) from the age of 16, but the combination therapy was discontinued because of fatigue. LDL-C: low-density lipoprotein cholesterol; TC: total cholesterol; HDL-C: high-density lipoprotein cholesterol; TG: triglycerides; AST: aspartate transaminase; ALT: alanine transaminase; E: Ezetimibe (10 mg), K: Kanuma (40 mg), R: Rosuvastatin (10 mg).

Before treatment, the mean height percentile of the children was 14.3 ± 5.7, and the mean BMI percentile was 30.4 ± 16.7. The children were not malnourished, but an improvement was seen in the height and BMI percentiles after treatment initiation. The mean percentile of height after treatment was 22.6 ± 3.2, and the mean BMI percentile was 45.9 ± 10.6. Ezetimibe (10 mg) was administered to all three children in the initial phase (Figure 2).

At age 10.4, patient 3 started receiving combination therapy with ezetimibe (10 mg) and Kanuma^®^ (1 mg/kg, every 2 weeks) in a clinical trial abroad. In 2017/2018, there was an interruption for 8 months with Kanuma^®^, because of organizing the therapy at a home institution, after discontinuing his treatment abroad. For patient 3, we noticed a decrease in TC, LDL-C, AST, ALT, and yGT levels on ezetimibe treatment alone, but a more important decrease is seen when Kanuma was added to the therapy, as seen in Supplementary Table S1. At age 16.8 in patient 1, no sufficient lowering of cholesterol and liver enzymes with ezetimibe was present at first and was not applicable for the Kanuma treatment, started with a combination of ezetimibe (10 mg) and rosuvastatin (10 mg). Moreover, the patient already had signs of endothelial dysfunction. We concluded that statins would be more beneficial to the patient because of persistently elevated cholesterol levels, but the patient had side effects (muscle pain and fatigue), and rosuvastatin was discontinued.

The levels of TC, LDL-C, HDL-C, TG, AST, and ALT levels in all three LAL-D children over time are presented in Figure 2. Vertical lines indicate therapy initiation and modification dates for all three children. Before the treatment, the mean AST and ALT levels were 1.33 ± 0.25 μkat/L and 1.61 ± 0.15 μkat/L, respectively. After treatment initiation, a decrease in the mean AST (0.73 ± 0.11 μkat/L) and ALT (1.13 ± 0.23 μkat/L) levels were observed. In the lipid profile after treatment, more favorable mean levels were present. The mean levels of TC (6.46 ± 0.95 mmol/L), LDL (4.34 ± 0.54 mmol/L), and triglycerides (1.44 ± 0.66 mmol/L) before treatment were higher than the mean TC (5.25 ± 0.40 mmol/L), LDL (3.38 ± 0.56 mmol/L), and triglycerides (1.16 ± 0.41 mmol/L) on treatment. Furthermore, HDL (1.29 ± 0.25 mmol/L) before treatment has increased on treatment (1.37 ± 0.33 mmol/L).

Chitotriosidase levels before treatment are only available in patient 2; the value was 408 nmol/mLh (reference range: 3–65 nmol/mLh). Comparing the first half (earlier period) of treatment with the second half (last period) of treatment in patients 2 and 3, we can see a decrease in chitotriosidase levels (from 426 ± 148 nmol/mLh to 300 ± 159 nmol/mLh in patient 2 and from 578 ± 197 nmol/mLh to 388 ± 57 nmol/mLh in patient three). In patient 1, the chitotriosidase levels were elevated in the second half of treatment; the values went from 654 ± 209 nmol/mLh to 682 ± 239 nmol/mLh.

Liver MRI in all children detected an enlarged liver but a normal-sized spleen. Mild fibrosis and steatosis of the liver in patients 1 and 2 were observed on MRI liver elastography, and in patient 3, high fibrosis in regions five and six has been present. The size of the liver in child 1 at 12.3 years was in the midclavicular plane in the craniocaudal line 159 mm and transverse line 185 mm. The degree of steatosis was 18%-19%. In the control after 4.5 years, the degree of steatosis was 10.2%. The size of the liver in patient 2 at 9.8 years was in the midclavicular plane in the craniocaudal line at 122 mm and transverse line at 190 mm, and the degree of steatosis was 16%-17%. In the control after 2.5 years, the degree of steatosis was 11%. The size of the liver in patient 3 at 10.2 years was in the midclavicular plane in the craniocaudal line at 148 mm and transverse line at 184 mm, and the degree of steatosis was 10%. In the control after 6.5 years, the degree of steatosis was 7%–10%. In all children, the spleen structure was normal on MRI elastography.

The mean cIMT was 0.353 ± 0.08 mm. By the references according to Drole Torkar et al. (2020), in patient 2, cIMT was between 25. and 50. p, and in the other two patients, it was under 5. p. The mean beta stiffness was 3.5 ± 0.5. By the references according to Doyon et al. (2013), patient 1 and patient 2 had beta stiffness above 50. p, and patient 3 under 50. p. The mean RHI was 1.38 ± 0.38. Using the threshold 1.35, suggested by Bonetti et al. (2004) classified patient 1 as having already endothelial disfunction, while patients 2 and 3 had a normal endothelial function. The mean PWV was 3.94 ± 0.46 m/s. By the references according to Reusz et al. (2010), patient 1 had PWV under the 5. p, and patients 1 and 2 were classified between 10. and 50. p.

### LAL-D Prevalence Estimation

The LAL-D prevalence in our population based on the data of heterozygous carriers of the pathogenic alleles in the *LIPA* gene was estimated at 1/406,193.

## Discussion

In the present study, we indicated the importance of the FH screening for the detection of dyslipidemia clinically expressed similarly to FH. As a result of having unspecific symptoms, LAL-D is diagnosed late in life, misdiagnosed, or overlooked. However, we aimed to distinguish between the phenotype of the children with FH and children with a *LIPA* disease-causing variant in our cohort. Furthermore, the prevalence of LAL-D in our population was estimated.

Cascade FH screening was successfully implemented in the Netherlands (Wiegman et al., 2015). Furthermore, as a great advantage of the universal hypercholesterolemia screening in preschool children (Groselj et al., 2018), in the absence of other FH monogenic or polygenic factors, simultaneously other dyslipidaemias could be detected. Moreover, LAL-D screening should be considered for children and young adults with unexplained hepatic elevated AST/ALT levels in combination with elevated LDL-C (>160 mg/dl, 4.1 mmol/L) and low HDL-C levels (<35 mg/dl, 1.0 mmol/L). Also, by universal FH screening, the LAL-D positive children are diagnosed, and appropriate treatment at an early age before the onset of serious clinical signs and disease progression is introduced (Strebinger et al., 2019). Another aim of early treatment is also the prevention of potential later cardiovascular complications as in one of our three children endothelial dysfunction was probably already present, considering that the RHI value was below the RHI cut-off value for adults (1.35) and lower RHI seen in the children from the same age group (Kelly et al., 2014).

All three children were homozygous for a pathogenic NM_000235.4: c.894G > A variant in the *LIPA* gene (NG_008194.1), which was previously functionally characterized as affecting a 5′ splice-region causing deletion of a 72-base exon 8 (p.Ser275_Gln298del) from the mRNA for LAL causing CESD letting 5%–10% of the wildtype activity (Klima et al., 1993; Ameis et al., 1995; Fasano et al., 2012). The presence of the c.894G > A variant is associated with a milder clinical phenotype of LAL-D with slow progression of liver fibrosis and cirrhosis (Lipiński et al., 2018). Rashu et al. (2020) describe two adult *LIPA* compound heterozygous siblings for the c.894G > A and c.482del variants and Gürbüz et al. (2020) report a 14-year-old and 3-year-old siblings with homozygous c.894G > A variant with persisting gastrointestinal symptoms (hepatosplenomegaly, malabsorption, and diarrhea, combined with elevated transaminases and dyslipidemia). A Columbian boy was confirmed with LAL-D with the c.893G > A variant in the *LIPA* gene at age 14 after isolated hepatomegaly and dyslipidemia at age 6 were detected (Botero et al., 2018).

LAL-D is an autosomal recessive disease since it has been demonstrated that heterozygous *LIPA* disease-causing carriers generally do not express an FH phenotype, while FH is a disease with an autosomal dominant inheritance pattern (Sjouke et al., 2016). The prevalence of LAL-D was previously estimated to be 1/40,000 to 1/300,000 individuals (Pullinger et al., 2015; Camarena et al., 2017). Based on the cohort of 1,915 children from our population the prevalence of homozygous LAL-D was estimated to be 1/1/406,193. Since we only considered pathogenic variants for the genetic computation of prevalence, rather than VUS, the prevalence in our population was slightly lower than expected compared to the literature.

Due to the unspecific symptoms, LAL-D is typically diagnosed late in life, misdiagnosed, or overlooked (Strebinger et al., 2019). In CESD, deaths because of liver disease progression have been reported already at the age of 7 years (Bernstein et al., 2013). Typically, children with LAL-D have elevated cholesterol levels, whereas the parents of the index case have normal lipid profiles (Sjouke et al., 2016). An important indicator for diagnosing children with LAL-D is significantly elevated transaminase levels, which is a sensitive and specific way of detecting this disorder. However, as screening for FH with cholesterol is already implemented in Slovenia, measuring TC levels at the first step and liver enzymes at the specialist clinic was more feasible. Although LAL-D should still be considered a differential diagnosis in children with increased liver enzymes or other clinical signs indicative of LAL-D. Moreover, LAL-D newborn screening (NBS) should be considered in the future (Remec et al., 2021); nevertheless, LAL-D currently is not screened in Europe and elsewhere by NBS (Loeber et al., 2021).

Moreover, statin therapy reduces LDL-C levels, but on the other hand, they promote the delivery of cholesteryl esters to hepatocytes and consequently worsens the effects on liver function (Pullinger et al., 2015; Sjouke et al., 2016). Liver transplantation has not been proven as a successful treatment option for LAL-D. Bernstein et al. (2013) reported that 11 out of 18 LAL-D children had multisystemic progression of the disease (for example, growth failure, anemia, sepsis, transplant rejection, right lower lobe collapse, atelectasis, pulmonary lipid deposition, dyslipidemia, atherosclerosis, and heart failure). Hematopoietic stem cell transplantation seems to be a more successful treatment in Wolman disease than liver transplantation, but there are also reports about disease progression and fatal transplant-related complications (Strebinger et al., 2019).

The use of a human recombinant LAL (sebelipase alpha, Kanuma^®^) for the LAL-D treatment was approved in 2015, not only resulting in significant improvements in the hepatic and lipid profiles of children with LAL-D but also increased survival rates in infants with Wolman disease (Sjouke et al., 2016; Camarena et al., 2017). In the results of the ARISE study, improvements in liver enzymes and lipid profile were seen with the Kanuma^®^ treatment. At the last open-label assessment, ALT and AST normalization were achieved by 47% and 66% of children, respectively. A 25% reduction in median (IQR) percent changes in LDL-C and a 27% (19%, 44%) increase in high-density lipoprotein cholesterol in the median (IQR) percent changes were observed (Burton et al., 2022). In 13 children who experienced an infusion-associated reaction, one was categorized as serious; the others were mild to moderate. Antidrug antibodies were found in 6 children in the middle of treatment, but in the end, 5 out of 6 children tested antidrug antibody negative, and the only one that was still positive was the patient that had developed activity-neutralizing antibodies (Burton et al., 2022). Although in 2017 in the National Institute for Health and Care Excellence (NICE) guidelines Kanuma^®^ was not recommended for long-term enzyme replacement therapy for treating LAL-D in babies with rapidly progressive disease and also not in children and adults (National Institute for Health and Care Excellence, 2017) since 2021 NICE is proceeding with a new Highly Specialised Technologies Evaluation for sebelipase (National Institute for Health and Care Excellence, 2021). In all three children from our study, an improvement in cholesterol levels, liver enzymes, and also of steatosis of the liver has been seen after early treatment initiation.

In 2020, consensus recommendations from an international collaborative working group were published on the initial assessment and ongoing monitoring of LAL-D in children and adults (Kohli et al., 2020). International guidelines on the treatment of LAL-D are lacking. In 2012 (recruiting is still in progress), an international registry was started to improve the understanding of therapeutic interventions and their long-term effectiveness (Soll et al., 2019).

One of the present study’s important limitations was a delay in diagnosing LAL-D when compared to age at FH screening. As a part of the universal FH screening, TC levels at the primary-care level were measured at age 5-6, and the median age by the referral to our institution was 6.3 (IQR: 5.8–7.2) years. Before an expanded NGS panel for dyslipidemia containing the *LIPA* gene was established, genetic testing was first performed to exclude FH, and therefore, genetic testing for LAL-D was performed later. After 2018, *LIPA* sequencing is performed on all FH screening positive children. Therefore, we do not anticipate any further delays in the future detection of the pathogenic variants in the *LIPA* gene.

In conclusion, our results show that universal FH screening in children is effective also for simultaneous identification of children with other rare dyslipidemia manifesting with hypercholesterolemia (such as LAL-D), as secondary screening conditions. An important clinical criterion for differentiation between FH and the LAL-D positive children was shown to be elevated levels of transaminases (ALT and AST). Early detection and treatment of children with LAL-D are important to prevent long-term consequences.

## Data Availability

The datasets presented in this study can be found in online repositories. The name of the repository and link to the data can be found: Mendeley; https://data.mendeley.com/datasets/zn7b85hgk6/1.
