# Supplementary material for: Early Discovery of Children With Lysosomal Acid Lipase Deficiency With the Universal Familial Hypercholesterolemia Screening Program
Source: Front Genet. 2022 Jul 12;13:936121. doi: 10.3389/fgene.2022.936121 (PMC9314654; doi:10.3389/fgene.2022.936121)
Supplement: Supplementary file 1 [file Presentation1.pdf]

Supplementary Material

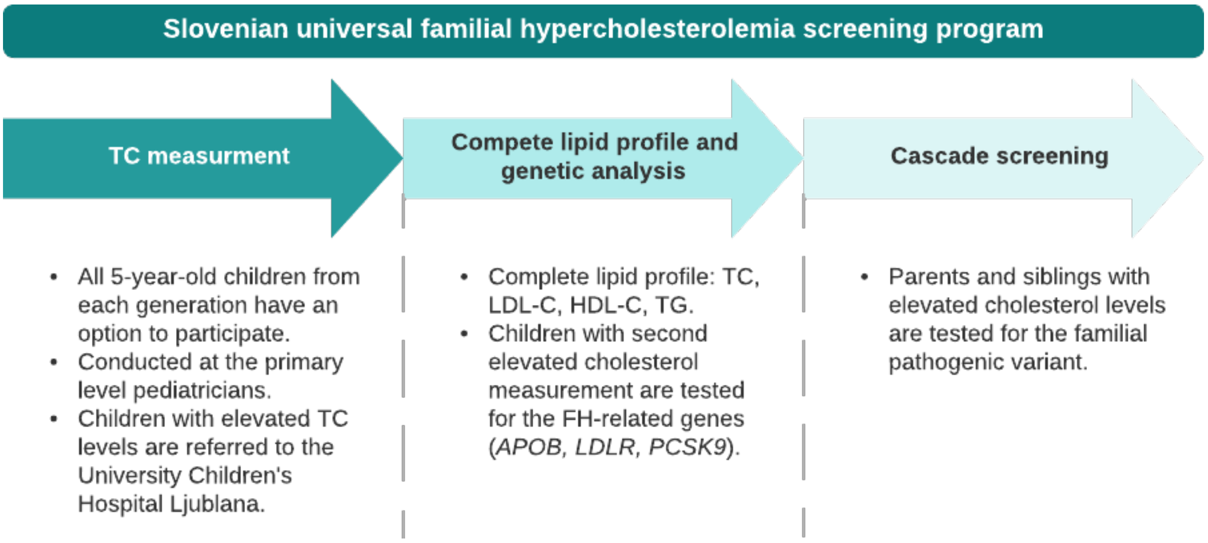

Supplementary Figure 1: Slovenian universal familial hypercholesterolemia (FH) screening program

Slovenia has been implementing universal FH screening in children since 1995 as a routine part of the blood check-up at the scheduled visit of all 5-year-old children to the primary care paediatrician, recently reaching more than 90% of the 5-year-old children (of approximately 20,000) each year (Sedej et al., 2014; Klancar et al., 2015; Groselj et al., 2018, 2022). Total cholesterol (TC) was assessed as the first step of the universal FH screening program. Children with elevated TC levels were referred to the University Children's Hospital Ljubljana for further investigation. Another lipid profile was taken and routine genetic testing for the children with elevated cholesterol levels for the FH-related genes (*APOB, LDLR, PCSK9*) was established in 2011. In 2019, we expanded the genetic panel to 18 genes, associated with dyslipidemia. In accordance with our clinical recommendations from 2019 extra cascade screening of family members with elevated cholesterol levels is performed.

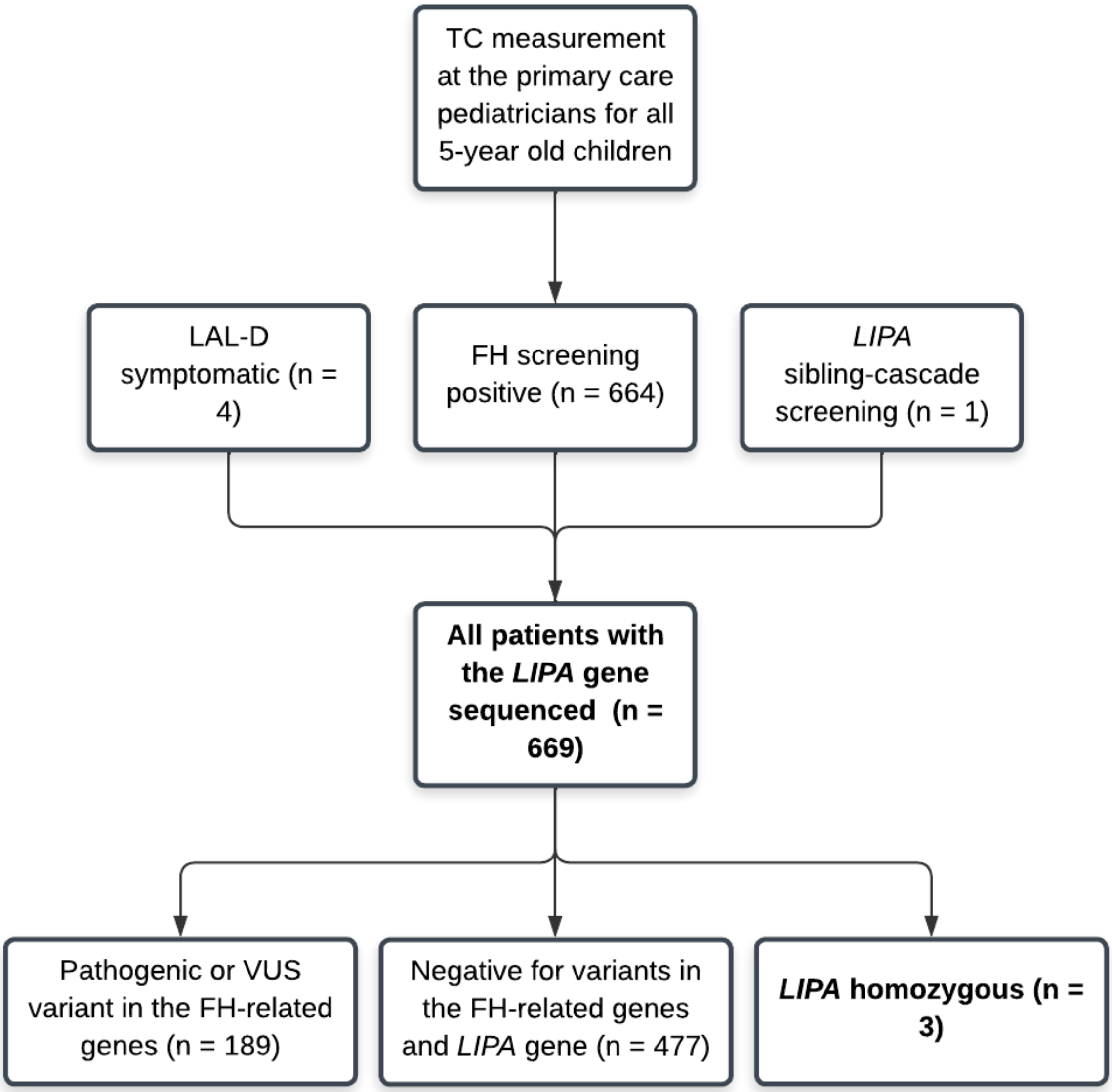

Supplementary Figure 2: Flowchart of the participants

As a part of the Slovenian universal FH screening program, 664 children with elevated total cholesterol (TC) levels were referred to the Univesity Children's Hospital Ljubljana. 4 children were referred for other reasons and had elevated liver transaminase levels, and were therefore included in our study. One child was referred later as a part of *LIPA* sibling-cascade screening. The total number of children genetically tested for pathogenic variants in the FH-related genes (*APOB, LDLR, PCSK9*) and in the *LIPA* gene was 669. 189 children had a pathogenic variant or variant with uncertain significance (VUS) in the FH-related genes. 447 children were negative for the variants in the FH-related genes and in the *LIPA* gene. Three children were homozygous for a pathogenic variant in the *LIPA* gene- 2 were referred from a Slovenian universal FH screening program and one as a part of the sibling-cascade screening.

Supplementary Table 1: Data on cholesterol and liver enzyme levels before and on treatment in patient 3

	Before treatment	Ezetimibe	Ezetimibe + Kanuma
**TC (mmol/L and (mg/dL))**	7.55 ± 0.84 (292 ± 32)	6.80 ± 0.28 (263 ± 11)	5.11 ± 0.77 (198 ± 30)
**LDL-C (mmol/L and (mg/dL))**	5.83 ± 0.74 (226 ± 29)	5.00 ± 0.28 (193 ± 11)	3.37 ± 0.77 (130 ± 30)
**HDL-C (mmol/L and (mg/dL))**	1.13 ± 0.20 (44 ± 8)	1.30 ±0.14 (50 ± 5)	1.18 ± 0.09 (46 ± 3)
**TG (mmol/L and (mg/dL))**	1.19 ± 0.35 (105 ± 31)	1.20 ± 0.28 (106 ± 25)	1.25 ± 0.31 (111 ± 28)
**AST (μkat/L)**	1.61 ± 0.38	0.86 ± 0.18	0.60 ± 0.17
**ALT (μkat/L)**	1.55 ± 0.34	1.12 ± 0.30	0.87 ± 0.33
**yGT (μkat/L)**	0.34 ± 0.07	0.30 ± 0.04	0.26 ± 0.07

TC (total cholesterol), LDL-C (low-density lipoprotein cholesterol), HDL-C (high-density lipoprotein cholesterol), TG (triglycerides), AST (aspartate aminotransferase), ALT (alanine aminotransferase) and yGT (gamma-glutamyltransferase) levels for the patient 3 before treatment (6 visits), treated only with ezetimibe (2 visits) and treated with ezetimibe and Kanuma (15 visits).

Supplementary References

Groselj, U., Kovac, J., Sustar, U., Mlinaric, M., Fras, Z., Podkrajsek, K. T., et al. (2018). Universal screening for familial hypercholesterolemia in children: The Slovenian model and literature review. *Atherosclerosis* 277, 383–391. doi: 10.1016/j.atherosclerosis.2018.06.858.

Groselj, U., Wiegman, A., and Gidding, S. S. (2022). Screening in children for familial hypercholesterolaemia: start now. *European Heart Journal*. doi: 10.1093/eurheartj/ehac224.

Klancar, G., Kovac, J., Bratina, N., Bratanic, N., Trebusak Podkrajšek, K., Groselj, U., et al. (2015). Universal Screening for Familial Hypercholesterolemia in Children. *J Am Coll Cardiol* 66, 1250–1257. doi: 10.1016/j.jacc.2015.07.017.

Sedej, K., Kotnik, P., Avbelj Stefanija, M., Groselj, U., Sirca Campa, A., Lusa, L., et al. (2014). Decreased prevalence of hypercholesterolaemia and stabilisation of obesity trends in 5-year-old children : possible effects of changed public health policies. *Eur J Endocrinol* 170, 293–300. doi: 10.1530/EJE-13-0566.
